# Acknowledgment of Reviewers

**DOI:** 10.1128/mBio.00413-20

**Published:** 2020-03-24

**Authors:** Arturo Casadevall

**Affiliations:** a Johns Hopkins University School of Medicine, Baltimore, Maryland, USA

## EDITORIAL

On behalf of the editors of *mBio*, I gratefully acknowledge the following individuals, who served as reviewers for the journal in 2019. Their time and effort in reviewing articles have been essential to ensuring the high quality of our publications, and their help is greatly appreciated.

Jacqueline Abranches
Gerhard Adam
Mark Adams
Michael Adams
Luis Agosto
Hiroji Aiba
Christopher Aiken
Klaus Aktories
Alexandre Alanio
Michaeline Albright
Felicity Alcock
John Alcorn
Bree Aldridge
Grace Aldrovandi
Alexander Alekseyenko
David Alland
Lee-Ann Allen
Luke Allsopp
Eric Alm
Alexandre Almeida
Francis Alonzo III
Craig Altier
Tomer Altman
Roberto Amato
Richard Ambinder
Jan Amend
Ricardo Amils
Ariel Amir
Kirk E. Anderson
Roy Anderson
David Andes
Alex Andrianopoulos
Hiroyuki Arai
Manuel Aranda
Nathalie Arhel
Cesar Arias
Chelsie Armbruster
David Armitage
Michelle Arnold
Cecilia Arraiano
Philip Ashton
David Askew
Haruyuki Atomi
Shannon Wing-Ngor Au
Yvonne Azasi
Paul Babitzke
Steffen Backert
Zeynep Baharoglu
Yong-Sun Bahn
Joel Baines
Susan Baker
Abhijeet Bakre
Matt Ballinger
Elizabeth Ballou
James Bangs
Abhisheka Bansal
Fernando Baquero
Alan Barbour
Amy Barczak
Sonia Bardy
Ralph Baric
Christopher Barnes
Michael Baron
Jeremy Barr
Timothy Barraclough
Alan Barrett
Jeffrey Barrick
Randall Basaraba
Susan Baserga
Christine Bassis
Philippe Bastin
Erik Bathoorn
Anthony Baughn
Alain Baulard
Andreas Bäumler
Victoria Baxter
Barbara Bayer
Josh Beck
Anke Becker
Steven Beer
Marcel Behr
Jessica Belser
Jose Bengoechea
Gordon Bennett
Richard Bennett
Andrew Benson
Roeland Berendsen
Megan Bergkessel
Cornelia Bergmann
Ben Berkhout
Judith Berman
Kristen Bernard
Cécile Berne
Thomas Bernhardt
Ronnie Berntsson
Maria Celia Bertolini
Sonja Best
Steve Beverley
Charles Bevins
Jennifer Bhatnagar
Somanon Bhattacharya
Nicolas Biais
Kai Bischof
Markus Bischoff
William Bishai
Dieter Blaas
Ira Blader
David Blair
Steven Blanke
Jill Blankenship
Joseph Bliss
Danielle Blondel
Paul Blount
Monika Bokori-Brown
David Bolam
Matteo Bonazzi
Robert Bonomo
Alisdair Boraston
Seth Bordenstein
Marina Borisova
Steeve Boulant
Cara Boutte
Grant Bowman
Jeff Boyd
Patricia Bradford
Todd Bradley
Axel Brakhage
Kristian Brandt
Catherine Braun-Breton
Miriam Braunstein
Richard Brennan
Bruce James Brew
Volker Briken
Melinda Brindley
Margo Brinton
Rebecca Brocato
Matthias Brock
Nichole Broderick
Konstantin Brodolin
Felix Broecker
Michael Bromley
Roland Brosch
Melissa Brown
Pamela Brown
Petr Broz
Eric Bruger
John Brumell
Wolfram Brune
Vincent Bruno
Nicole Buan
Juliane Bubeck Wardenburg
Shilpa Buch
Johannes Buchner
Carmen Buchrieser
Daniel Buckley
Angus Buckling
Nienke Buddelmeijer
Bernd Bukau
Dmitry Bulavin
Rebecca Burgess
Gaelen Burke
Vincent Burrus
Karen Bush
David Bzik
Matthew Cabeen
Ken Cadwell
Michael Calcutt
Ben Callahan
Jodi Camberg
Terri Camesano
Andrew Camilli
David Campbell
Kenneth Campellone
J. Gregory Caporaso
Massimo Caputi
Hannah Carey
Paul Carini
Yehuda Carmeli
Mark Carrington
Carol Carter
Nadia Casillas Ituarte
Jim Cassat
Clayton Caswell
Amy Caudy
Michael Chi Wai Chan
Yung-Fu Chang
John Chaston
Dhruba Chattoraj
Changbin Chen
Chaoping Chen
Liang Chen
Swaine Chen
Xian-Ming Chen
Sylvie Chevalier
Nicholas Chia
Peter Chien
Abhilash Chiramel
Byung-Kwan Cho
Jang-Cheon Cho
George Christophides
Oana Ciofu
Daniela Cirillo
Geremy Clair
Catherine Clarke
Ian Clarke
Thomas Clarke
Martha R. J. Clokie
N. G. Cogan
Eric Cohen
Gary Cohen
Taylor Cohen
Laurie Comstock
Ciaran Condon
Brian Conlon
John Connor
Gregory Cook
Tim Cooper
Teresa Coque
Pierre Cornelis
Mireia Coscolla
Kyle Costa
Ernesto Cota
Mathieu Coureuil
Russell Cox
Katharine Coyte
Robert Cramer
Brian Crane
Peter Crompton
Sean Crosson
Laszlo Csonka
Bryan Cullen
Valeria Culotta
Carole Dabney-Smith
Ross Dalbey
Angela Dale
Blossom Damania
Pranav Danthi
Isabelle Darboux
Sophie Darch
K. Darwin
Mary Davey
Lawrence David
Michael David
Andrew Davidson
Bryan Davies
Mark Davies
Kasey Day
Kristen DeAngelis
Laurent Debarbieux
Piet de Boer
James DeCaprio
Luiz Pedro de Carvalho
Christophe de Champs
Patrick Degnan
Kirk Deitsch
Juan de la Torre
Frank DeLeo
Jean-Sebastian Delisle
Maurizio Del Poeta
Neal DeLuca
Diego de Mendoza
Liesbeth Demuyser
Erick Denamur
Tanneke den Blaauwen
Keith Derbyshire
Zygmunt Derewenda
David DeShazer
Devyani Deshpande
Rik de Swart
Corrie Detweiler
Gill Diamond
Michael Diamond
Gregory Dick
Andreas Diepold
Matthew Dietz
Stephen Diggle
Jimmy Dikeakos
Siyuan Ding
Victor DiRita
Dirk Dittmer
Thuy Do
Ulrich Dobrindt
Kathryn Docherty
David Dockrell
Yohei Doi
Min Dong
Tao Dong
Anna Dongari-Bagtzoglou
Tobias Dörr
Zhicheng Dou
Angela Douglas
Lorenzo Drago
Christian Drosten
Rebecca Drummond
Rebbeca Duar
David Dubnau
Breck Duerkop
Iain Duggin
Sylvia Duncan
Julie Dunning Hotopp
Rebecca Dutch
Jeffrey Dvorin
Jonathan Dworkin
Michelle Dziejman
Mira Edgerton
Yoko Eguchi
Michael Ehrmann
Bart Eijkelkamp
Shirit Einav
Jonathan Eisen
Michael Eisenhut
Eugenin Eliseo
Craig Ellermeier
Marie Elliot
Sean Elliott
Mohamed El-Naggar
Lindsay Eltis
David Engelthaler
Richard Engh
David Engman
Markus Engstler
Luis Enjuanes
Jost Enninga
Lynn Enquist
Alexander Ensminger
Hyungjin Eoh
A. Murat Eren
Marc Erhardt
Madeleine Ernst
Peter Espenshade
David Evans
Matthew Evans
Federica Facciotti
Oliver Fackler
Ferric Fang
Mingxu Fang
Mark Farman
Michael Farzan
Guido Favia
Michael Federle
Michael Feldbrügge
Mario Feldman
Heinrich Feldmann
Hanping Feng
Honglin Feng
Zongdi Feng
Paul Fey
David Fidock
Kenneth Fields
Noah Fierer
Laura Filkins
Alain Filoux
Steven Finkel
J. Ross Fitzgerald
Kathryn Fixen
Klas Flardh
Enrique Flores
Ervin Fodor
Anja Forche
Robert Ford
Katrina Forest
James Forrest
Konrad Förstner
Elizabeth Fortunato
Jarrod Fortwendel
Kevin Foster
Patricia Foster
Betsy Foxman
Gad Frankel
Kurt Fredrick
Stephen Free
Michael Freitag
Nancy Freitag
Eva Frickel
Harvey Friedman
Timothy Friesen
Heather Fritz
Barbara Funnell
Sarah Gaffen
Kenneth Gage
Sara Gago
Tom Gallagher
Benoît Gamain
Robert Garcea
Joe G. N. Garcia
Ricardo Garcia
Francisco García-del Portillo
Jaime Garcia-Mena
Ethan Garner
Ruben Garrido-Oter
Danielle Garsin
Beth Garvy
Josep Gasol
Beilei Ge
Timothy Geary
Harris Gelbard
Mikhail Gelfand
Angie Gelli
Aleeza Gerstein
Benjamin Gewurz
Rohit Ghai
Mahmoud Ghannoum
David Giedroc
Jason Gigley
Tim Gilberger
Nicole Gilbert
Tom Gilbert
Daniel Gillet
Paul Gilson
Zemer Gitai
Amy Gladfelter
Pierre Gladieux
Travis Glare
Jane Glazebrook
Michael Glickman
Henry Godfrey
Michel Gohar
Daniel Goldberg
Marcia Goldberg
Erin Goley
Michael Good
Felicia Goodrum
Heinrich Gottlinger
Friedrich Götz
Mark Goulian
Marcin Grabowicz
Carly Grant
Peter Graumann
Justin Greene
Warner Greene
Finn Grey
Sergio Grinstein
Carol Gross
Martin Grube
Niklaus Grunwald
Haidong Gu
Lan Guan
John Guatelli
Marc-Jan Gubbels
Nancy Guillen
Reto Guler
Keith Gull
John Gunn
Arthur Günzl
James Gurney
Lionel Guy
Georg Häcker
Michael Habig
Ted Hackstadt
Maria Hadjifrangiskou
Matthias Hahn
Torsten Hain
Mohamed Hakimi
Rebecca Hall
Richard Hamelin
Marie-Louise Hammarskjold
Brian Hammer
Neal Hammer
Christopher Hammerbeck
Leendert Hamoen
Lynn Hancock
Jo Handelsman
James Hane
Cara Haney
Bishoy Hanna
Thomas Hannan
Klaus Hantke
Siegfried Hapfelmeier
William Harcombe
Andrew Harman
Joe Harrison
Jörg Hartig
Eric Harvill
Asma Hatoum-Aslan
Manajit Hayer-Hartl
Christopher Hayes
Cynthia He
Jizheng He
Aziz Heddi
Lars Hederstedt
Brian Hedlund
Jurgen Heesemann
Christine Heilmann
Franz Heinz
Robert Heinzen
Mark Heise
Joseph Heitman
Sophie Helaine
John Helmann
Michael Hensel
Scott Hensley
Georges Herbein
Christian Herrmann
Anat Herskovits
Erik Hewlett
Meleah Hickman
Colin Hill
N. Luisa Hiller
Ya-Chi Ho
Allon Hochbaum
Anne Hoen
Paul Hoffman
Deborah Hogan
Tobias Hohl
David Holden
Edward Holmes
Erik Holmqvist
Stacy Horner
Curt Horvath
Benjamin A. Horwitz
Paul Hoskisson
Jaroslav Hrabak
Elaine Hsiao
Yilin Hu
Victor Huber
Philip Hugenholtz
Grant Hughes
Kelly Hughes
Bruce Hungate
Bryan Hurley
Greg Hurst
Laurence Hurst
Terence Hwa
Geelsu Hwang
Nathalien S. Hwang
Joseph Hyser
Michael Ibba
Ashraf Ibrahim
James Imlay
Anne Immberty
Hideo Iwai
Akiko Iwasaki
Mary Ann Jabra-Rizk
Mary Jackson
William Jackson
William R. Jacobs, Jr.
Ilse Jacobsen
Alexander Jaffe
Elie Jaime
Tim James
Theodore Jardetzky
Katy Jeannot
Urs Jenal
Anja T. R. Jensen
Junhyun Jeon
Patric Jern
Mike Jetten
Peihua Jiang
Rays H. Y. Jiang
Yongqin Jiao
Francis Jiggins
Eric Johannsen
Michael Johnson
Welkin Johnson
Simon Johnston
Kristina Jonas
Howard Judelson
Jae Jung
Kato Junichi
Sheryl Justice
Praveen Juvvadi
Aras Kadioglu
Robert Kalejta
Marina Kalyuzhnaya
John Kao
James Kaper
Ronan Kapetanovic
Stefan Kappe
Petros Karakousis
Peter Karp
Stephanie Karst
Wali Karzai
Kazuyuki Kasahara
Fatah Kashanchi
Souichiro Kato
Alexis Kaushansky
Yoshihiro Kawaoka
Thomas Kawula
Joseph Keane
Mary Kearney
Daniel Kearns
Seung-Jung Kee
Thomas Kehl-Fie
Nancy Keller
Melissa Kendall
Peter Kennedy
Linda Kenney
Scott Kenney
Shannon Kenney
Stephen Kent
Anupama Khare
Margaret Kielian
Yoshitomo Kikuchi
Hyun Uk Kim
Joy Kim
Jun-Seob Kim
James Kirby
Adrienne Kish
Corby Kistler
Heather Kittridge
Stephanie Kivlin
Robyn Klein
Hans-Dieter Klenk
Kimberly Kline
Karl Klose
Stephen A. Klotz
Olaf Kniemeyer
Laura Knoll
Bruce Knutson
Katia Koelle
Gerwald Köhler
Ilana Kolodkin-Gal
Max Kolton
Ekaterina Koltsova
Anna Konovalova
Sebastian Kopf
Amnon Koren
Thomas Kornberg
Britt Koskella
Vassiliki Koufopanou
Reinhard Kraemer
Bernhard Kraeutler
Florian Krammer
Barry Kreiswirth
Laurent Kremer
Matthias Kretschmer
Christopher Kristich
James Kronstad
Damian Krysan
Ed Kuijper
Ashok Kumar
Rolf Kümmerli
Chih-Horng Kuo
Marcelo Kuroda
Jason Kwan
Kyung Kwon-Chung
Jason Kwong
Maurício Lacerda Nogueira
Tim Lachnit
Borden Lacy
Nadine Laguette
Laimonis Laimins
Seema Lakdawala
Francois Lallier
Robert Lamb
Richard Lamont
Tilman Lamparter
Kristin Lane
Andrew Lang
Michael Lanzer
Inigo Lasa
Jean-Paul Latgé
Adam Lauring
Thomas Lavsten
Beth Lazazzera
Brian Lazzaro
Chia Lee
Janet Lee
Margie Lee
Pushkar Lele
Cammie Lesser
Petra Levin
Kim Lewis
Chunhao Li
Jianrong Li
Renfeng Li
Shitao Li
Bruno Lima
Andrew Limper
Zhen Lin
Brett Lindenbach
Lisa Lindesmith
Steven Lindow
Hannes Link
Michail Lionakis
John LiPuma
Anastasia Litvintseva
Haoping Liu
Shan-Lu Liu
Xiong Liu
Manuel Llinas
Shawn Lockhart
Jennifer Lodge
Martin Loessner
Edmund Loh
Olga Lomovskaya
Torey Looft
Carolina B. López
Gloria Lopez-Castejon
Jose Lopez-Ribot
Anice Lowen
Ling Lu
Yahai Lu
Jeremy Luban
Aron Lukacher
Nicholas Lukacs
Ruth Ann Luna
Ming Luo
Meghan Lybecker
R. Craig MacLean
Juan Maestre
Suresh Mahalingam
Tomás Maira-Litrán
Michael Malamy
Matthew Malone
Peter Mancuso
Keerthi Mandyam
Michael Manson
Yuxin Mao
David Marchant
William Margolin
Mary Markiewicz
Luciano Marraffini
José Martínez
Miguel Martinez
Norma Martinez-Gomez
Nelson Martins
Sergei Maslov
Kevin Mason
Eric Massé
Ruth Massey
Kalai Mathee
Jyl Matson
Yasufumi Matsumura
Michael Maurizi
Wendy Maury
Harold May
Emeran Mayer
Laura Mazzaferro
Andrew McArthur
Linda McCarter
Mark McClain
Grant McClarty
Malcolm McConville
John McCutcheon
Sarah McDonald
Diane McDougald
Anita McElroy
James McKinlay
Rachel McLoughlin
Alan McNally
Reginald McNulty
Joan Mecsas
Conor Meehan
Andrew Mehle
Gregory Melikian
Ralf Mendel
Francie Mercer
Sabeeha Merchant
Janet Mertz
Ilhem Messaoudi
Michelle Meyer
Thomas Meyer
Eisha Mhatre
Jan Michiels
Francesca Micoli
Matthew Miller
Samuel Miller
Virginia Miller
Wolfgang Miller
Michael Mina
Tohru Minamino
Tiago Mineo
Rajeev Misra
Aaron Mitchell
Tim Miyashiro
Harry Mobley
Edward Mocarski
Attila Mocsai
Jennifer Moffat
Ian Mohr
Michelle Momany
David Montefiori
Cary Moody
Robert Mooney
Ann Moormann
Abraham Morales-Cruz
Gary Moran
Gonzalo Moratorio
Nicole Moreland
Iain Morgan
Yasu Morita
Koki Morizono
Lynn Morris
Robert Morris
Joachim Morschhäuser
Annika Mosier
Catherine Mounier
Christopher Moxon
W. Moye-Rowley
Jianbing Mu
Atis Muehlenbachs
Ryan Mueller
Ulrich Mueller
Liliane Mukaremera
Bela Mulder
Daniel Muller
Volker Müller
Mark Mulligan
Carsten Münk
Carol Munro
Erin Murphy
Kenan Murphy
Mario Muscarella
Alessandro Muzzi
Mojgan Naghavi
Keiichi Namba
Arshan Nasir
Carl Nathan
Daniel Neafsey
Stuart Neil
Daniel Nelson
David Nelson
Dianne Newman
Irene Newton
Caroline Ng
Timothy Nice
Wayne Nicholson
Robert Niederman
Kaare Magnus Nielsen
Christina Nielsen-LeRoux
Aleksandra Nita-Lazar
Justin Nodwell
Marcelo Nollmann
Jean-Philippe Nougayrede
Mairi Noverr
Minou Nowrousian
Torsten Ochsenreiter
Tamara O’Connor
Stefan Oehlers
Cliona O’Farrelly
Maya Ofek-Lalzar
Peter O’Hare
Michal Olszewski
Teresa O’Meara
Thomas Onchuru
Wai Kit Ong
Akira Ono
Eng Eong Ooi
Mary O’Riordan
David Ornelles
Eric Oswald
George O’Toole
Melanie Ott
Karen Otteman
Michael Otto
Scot Ouellette
F. Wayne Outten
Kelli Palmer
Bernhard Palsson
Maria-Eirini Pandelia
John Panepinto
Kai Papenfort
Rebecca Parales
Joanna Parish
Benjamin Parker
John Parker
Colin Parrish
Bruce Paster
Shelley Payne
Richard Peek, Jr.
Gang Pei
José Penades
Rafael Peña-Miller
Xuanxian Peng
Daniel Perez
Marcos Pérez-Losada
John Perfect
David Perlin
Lena Pernas
Jean-Luc Pernodet
Marc Peters-Golden
Sebastien Pfeffer
Caroline Philpott
Anne Piantadosi
Ameet Pinto
Javier Pizarro-Cerdá
Mariagrazia Pizza
Vicente Planelles
Alexander Pletnev
Mircea Podar
Stefanie Pöggeler
Claudia Pogoreutz
Stefan Pöhlmann
Mechthild Pohlschroder
Patrice Polard
Tino Polen
Leo Poon
Daniel Portnoy
Spyros Pournaras
Fanny Pouyet
Evan Powers
Lance Price
Alice Prince
Amy Pruden
Michael Pucci
Dohun Pyeon
Weigang Qiu
Xiangguo Qiu
Joseph Qualls
Robert Quivey
Nancy Raab-Traub
Korneel Rabaey
Samuel Rabkin
Elze Rackaityte
Simona Radutoiu
Manuela Raffatellu
Stephen Ragsdale
Tracy Raivio
Seth Rakoff-Nahoum
Lalita Ramakrishnan
Kumaran Ramamurthi
Joshua Ramsay
David Rasko
Angela Rasmussen
John Rawls
Daniel Read
Robert Read
Sylvie Rebuffat
Michael Reese
R. Keith Reeves
John Regan
Larry Reitzer
Han Remaut
Michelle Reniere
Christopher Rensing
Blanca Restrepo
Federico Rey
Anna-Louise Reysenbach
Jose Ribeiro
Andrew Rice
Scott Rice
Gregory Richards
Tom Richards
Anthony Richardson
Martin Richer
Michael Riscoe
Douglas Risser
Amariliz Rivera
Marilyn Roberts
Derrick Robinson
Eduardo Rocha
Charles Rock
Francisco Rodriguez-Valera
Tony Romeo
Ute Römling
Ze’ev Ronai
Lijun Rong
Diana Roopchand
Jason Rosch
Andrew Rose
Eugene Rosenberg
Amy Rosenfeld
Ashley Ross
Susan Ross
Amelia-Elena Rotaru
Michael Rother
Leah Rubin
Thomas Rudel
Gloria Rudenko
Stefan Rudiger
Paolo Ruggerone
Tige Rustad
Juliana Sá
Zakee Sabree
Jeroen Saeij
Jeanne Salje
Dor Salomon
Robert Samson
John Samuelson
Marina Sánchez-Hidalgo
Jon Sanders
Rozanne Sandri-Goldin
Dominique Sanglard
Álvaro San Millan
Thomas Santangelo
Eduardo Santero
Alfonso Santos-Lopez
Frank Sargent
Carlos Sariol
Christopher Sassetti
Karla Satchell
Quentin Sattentau
Uwe Sauer
Daniel Sauter
Barry Saville
Jimmy Saw
Gary Sawers
Luis Schang
Birgit Scharf
Dirk-Jan Scheffers
Tilman Schirmer
Michael Schloter
Amy Schmid
Eric Schmidt
Alexa Schmitz
Stephan Schmitz-Esser
Monika Schmoll
Dirk Schnappinger
David Schneider
Karin Schnetz
Peter Schönheit
Tony Schountz
Jared Schrader
Matthew Schrenk
Gunnar Schroeder
Torsten Schubert
Klaus Schughart
Stacey Schultz-Cherry
Maria Schumacher
Edward Schwartz
Egbert Schwartz
Robert Schwartz
Jodi Schwarz
Herbert Schweizer
Martin Schwemmle
Robert Seder
Jessica Seeliger
Kate Seib
Bert Semler
Diego Serra
Stephanie Seveau
Ashley Shade
Justin Shaffer
Sasha Shafikhani
Dhanasekaran Shanmugam
Jason Shapiro
Brian Shaw
Jessica Sheldon
Nathan Sherer
David Sherman
Barbara Sherry
Yixin Shi
Patrick Shih
Bo Shopsin
Masahiro Shuda
Jacqui Shykoff
Alex Sigal
Robert Siliciano
Rafael Silva-Rocha
Lyle Simmons
Viviana Simon
Amit Singhal
Albert Siryaporn
Eric Skaar
Rebecca Skalsky
James Slauch
Joe Smith
Wiep Klaas Smits
Miguel Soares
Jack Sobel
Thierry Soldati
Dominique Soldati-Favre
Greg Somerville
Holger Sondermann
Wenxia Song
Justin Sonnenburg
Joseph Sorg
Maria Soto
Victor Sourjik
Brad Spellberg
Katherine Spindler
Stephen Spiro
Alfred Spormann
Jason Stajich
Pierre Stallforth
Christina Stallings
Alfons Stams
Nicola Stanley-Wall
Bruce Stanton
Spyridon Stavrou
James Stegen
Marc Stegger
Lisa Stein
William Steinbach
Ionis Stergiopoulos
Silke Stertz
Brian Stevenson
Frank Stewart
Melissa Stockwell
Paul Stoodley
Gisela Storz
Klaus Strebel
Michael Strickland
Jorg Stulke
Agathe Subtil
Reiko Sugiura
Jung Soo Suk
David Sullivan
William Sullivan
Guangyu Sun
Weina Sun
Venkatesan Sundaresan
Michael Surette
Mehul Suthar
Richard Sutton
Nobuhiro Suzuki
Ronald Swanstrom
W. Edward Swords
David Sychantha
Christine Szymanski
Michiko Taga
K. P. Connie Tam
Rita Tamayo
Hengli Tang
Azuma Taoka
Vera Tarakanova
Phillip Tarr
Alice Telesnitsky
Italo Tempera
Tobias Tenenbaum
Benjamin tenOever
Casey terHorst
Herve Tettelin
Lenny Teytelman
Bo Thamdrup
David Thanassi
Martin Thanbichler
Patricia Thibault
T. Frede Thingstad
Andrew Thomas
Torsten Thomas
Bart Thomma
Justin Thornton
Leann Tilley
Anna Tischler
Till Tiso
Alexander Titz
Andrew Tobin
David Tobin
Elitza Tocheva
Makrina Totsika
Thierry Touzé
Steven Townsend
Frances Trail
Leonardo H. Travassos
Matthew Traxler
Todd Treangen
Yannick Tremblay
M. Stephen Trent
Brian Trevelline
Mirko Trilling
Brian Tsuji
Tomoya Tsukazaki
Kürsad Turgay
Marta Turon
Rodney Tweten
Hanne Tytgat
Takamasa Ueno
Nathan Ungerleider
Constantin Urban
Raphael Valdivia
Susana Valente
Vito Valiante
Sophie Valkenburg
Adrian Valli
Miguel Valvano
Peter van Baarlen
Bert van den Berg
Giel van Dooren
Stineke van Houte
Julia van Kessel
Steven Van Lanen
Laurence Van Melderen
Tim van Opijnen
Mark van Passel
Jan-Peter van Pijkeren
Daria Van Tyne
Gilles van Wezel
Raghavan Varadarajan
Michael Vasil
Subhash Vasudevan
Tommi Vatanen
Nora Vazquez-Laslop
Andres Vazquez-Torres
Jan-Willem Veening
David Veesler
Marco Ventura
Saguna Verma
Kevin Verstrepen
Alejandro Vila
Patrick Viollier
Karen Visick
Lucía Vivanco
Marnix Vlot
Joerg Vogel
Kevin Vogel
Christian Voolstra
Daniel Voth
Jovanka Voyich-Kane
Jatin Vyas
Lawrence Wackett
Joseph Wade
David Wagner
Maggie Wagner
Mark Walker
Zoe Waller
Jens Walter
David Wang
Jian-Ying Wang
Jue Wang
Ping Wang
Xiaofeng Wang
Yue Wang
Digby Warner
Christopher Waters
Michael Watson
Robert Watson
Nathaniel Weathington
Richard Webby
Mary Weber
Gunter Wegener
James Weger-Lucarelli
David Weiss
Louis Weiss
Matthew Weitzman
Matthew Welch
Sandra Weller
Melanie Wellington
Zoey Werbin
Christopher West
Edze Westra
Cynthia Whitchurch
Peter White
Marvin Whiteley
Katrine Whiteson
Malcolm Whiteway
John Whitney
Jelte Wicherts
Robert Wilkinson
Allison Williams
Peter Williamson
Tomasz Wilmanski
Kevin Wilson
Michael Wilson
Richard A. Wilson
Van Wilson
Sebastian Winter
Martin Witzenrath
Charles Woloshuk
Gary Wong
Thomas Wood
Christopher Woods
Floyd Wormley, Jr.
Daniel Wozniak
Rachel Wozniak
Erik Wright
Gavin Wright
Gerard Wright
Terry Wright
Kelly Wrighton
Hui Wu
Kristine Wylie
Jie Xiao
Chaoyang Xue
Timothy Yahr
Chung Yang
Jun Yang
Yu-Feng Yao
George Yap
Sara Yeo
Hasan Yesilkaya
Joanne Yew
Ryland Young
Andrew Yurochko
Boaz Yuval
Joseph Zackular
Chuanlun Zhang
Jingren Zhang
Lianhui Zhang
Shizhu Zhang
Wenjing Zhang
Youfu Zhao
Fanxiu Zhu
Hongyan Zhu
Jian Zhu
Jun Zhu
Joseph Ziegelbauer
Jochen Zimmer
Joshua Zimmerberg
Peter Zuber
Wolfram Zueckert

